# Supplementary material for: Drug repurposing for aging research using model organisms
Source: Aging Cell. 2017 Jun 16;16(5):1006–15. doi: 10.1111/acel.12626 (PMC5595691; doi:10.1111/acel.12626)
Supplement: Supplementary file 7 — Data S1 Zip‐Archive of all report cards. [file ACEL-16-1006-s007.zip › RC_CHD.pdf]

CHD

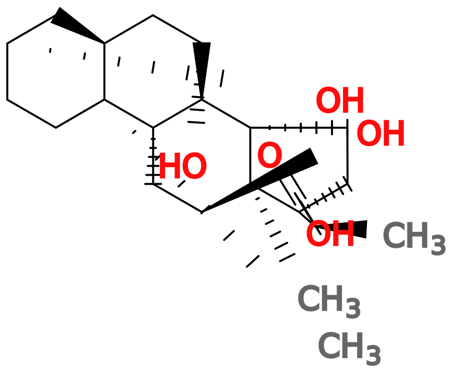

Database identifiers

ChEMBLCompound	CHEMBL205596
DrugBank	DB02659
CHEBI	16359
eMolecules	29504030
eMolecules	29541145
eMolecules	30512922

Ranking

	Rank	Score
Drosophila	515/697	0.253
C. elegans	NA	NA

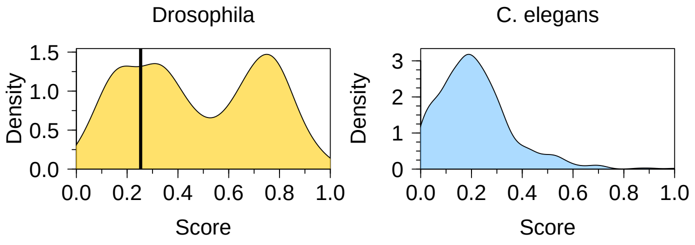

	Ageing implication	Domain conservation	Binding site conservation	Binding affinity	Bioavailability	Lipinski	Promiscuity	Purchasability	Drug approval	**Total**
Drosophila	1.0	0.591	0.614	0.241	(0.9)	0.0	-0.0	0.1	0.075	0.253
C. elegans	NA	NA	NA	NA	NA	NA	NA	NA	NA	NA

Names

- **cholic acid**
- (3alpha,5beta,7alpha,12alpha)-3,7,12-trihydroxycholan-24-oic acid
- 3alpha,7alpha,12alpha-Trihydroxy-5beta-cholanate
- 3alpha,7alpha,12alpha-Trihydroxy-5beta-cholanic acid
- Cholsaeure

Roles

human metabolite, mouse metabolite

Status

Approved drug (according to ChEMBL)	No
Classification (according to DrugBank)	experimental
Number of Rule of 5 violations	0
Binding affinity to original target in log units (RF-Score prediction)	3.85
Burns *C. elegans* bioavailability prediction	-10.87

Compound Target Characteristics

Menin

Best gene implication in ageing for this target family came from gene Q8SXU2 annotated in UniProt release 2014_02. Annotation GO 8340 (determination of adult lifespan) was Inferred from Mutant Phenotype

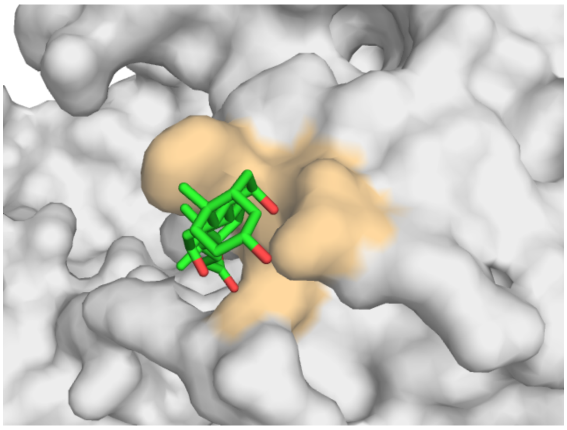

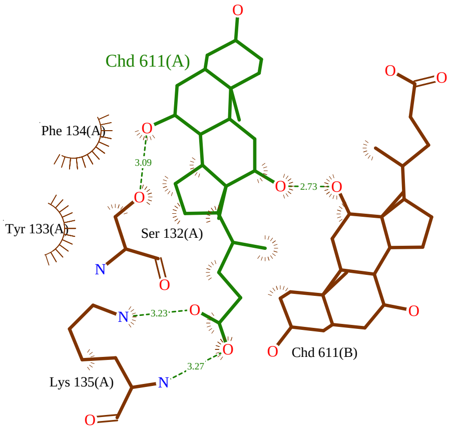

protein	amino acids contacts (binding site)
PDB:3u88:chainA:O00255	S Y F K
tr:Q9GZQ5:Q9GZQ5_HUMAN	S Y F K
tr:E7ENS2:E7ENS2_HUMAN	S Y F K
tr:E7EPR4:E7EPR4_HUMAN	S Y F K
tr:E7ET29:E7ET29_HUMAN	S Y F K
tr:E7EN32:E7EN32_HUMAN	S Y F K
sp:O00255:MEN1_HUMAN	S Y F K
sp:Q9WVR8:MEN1_RAT	S Y F K
tr:D3YZC2:D3YZC2_MOUSE	S Y F K
sp:O88559:MEN1_MOUSE	S Y F K
tr:F8WHD9:F8WHD9_MOUSE	S Y F K
tr:Q8SXU2:Q8SXU2_DROME	S S Y K
tr:Q7KXY9:Q7KXY9_DROME	S S Y K
tr:Q9VM47:Q9VM47_DROME	S S Y K

protein	whole protein ident	simil	domain-based ident	simil	contact-based ident	simil
PDB:3u88:chainA:O00255	1.0	1.0	1.0	1.0	1.0	1.0
tr:Q9GZQ5:Q9GZQ5_HUMAN	0.24	0.24	0.24	0.24	1.0	1.0
tr:E7ENS2:E7ENS2_HUMAN	0.42	0.42	0.43	0.43	1.0	1.0
tr:E7EPR4:E7EPR4_HUMAN	0.46	0.46	0.46	0.46	1.0	1.0
tr:E7ET29:E7ET29_HUMAN	0.56	0.56	0.56	0.56	1.0	1.0
tr:E7EN32:E7EN32_HUMAN	0.9	0.9	0.9	0.9	1.0	1.0
sp:O00255:MEN1_HUMAN	1.0	1.0	1.0	1.0	1.0	1.0
sp:Q9WVR8:MEN1_RAT	0.96	0.98	0.96	0.98	1.0	1.0
tr:D3YZC2:D3YZC2_MOUSE	0.9	0.92	0.9	0.92	1.0	1.0
sp:O88559:MEN1_MOUSE	0.96	0.98	0.96	0.98	1.0	1.0
tr:F8WHD9:F8WHD9_MOUSE	0.95	0.97	0.95	0.97	1.0	1.0
tr:Q8SXU2:Q8SXU2_DROME	0.36	0.64	0.37	0.65	0.5	0.61
tr:Q7KXY9:Q7KXY9_DROME	0.32	0.6	0.34	0.64	0.5	0.61
tr:Q9VM47:Q9VM47_DROME	0.32	0.59	0.34	0.64	0.5	0.61

Mnn1 (FBgn0031885) associated phenotypes

DNA repair defective, chemical sensitive, conditional, heat sensitive, lethal - all die before end of P-stage, melanotic mass phenotype, radiation sensitive, short lived, some die during pharate adult stage, stress response defective

(Information from FlyBase)
